# Effects of systemic ventricular assist in failing Fontan patients: a theoretical analysis using a computational model

**DOI:** 10.1186/s12576-024-00946-z

**Published:** 2024-11-02

**Authors:** Eiri Kisamori, Yasuhiro Kotani, Toshiaki Shishido, Shingo Kasahara, Shuji Shimizu

**Affiliations:** 1 Department of Cardiovascular Surgery, Okayama University Graduate School of Medicine, Dentistry, and Pharmaceutical Sciences and Okayama University Hospital, 2-5-1 Shikatacho, Kitaku, Okayama 700-8558 Japan; 2 Department of Research Promotion and Management, National Cerebral and Cardiovascular Center, 6-1 Kishibe-Shimmachi, Suita, Osaka 564-8565 Japan

**Keywords:** Ventricular assist device, Failing Fontan, Hemodynamic simulation, Lumped parameter model

## Abstract

Mechanical circulatory support is a potential treatment for failing Fontan patients. In this study, we performed a theoretical analysis using a computational model to clarify the effects of systemic ventricular assist device (VAD) in failing Fontan patients. Cardiac chambers and vascular systems were described using the time-varying elastance model and modified Windkessel model, respectively. A VAD was simulated as a nonlinear function. In systolic and diastolic ventricular dysfunction and atrioventricular valve regurgitation models, systemic VAD increased the cardiac index and decreased the central venous pressure (CVP). However, in the high pulmonary vascular resistance model, CVP became extremely high above 15 mmHg to maintain the cardiac index when the pulmonary vascular resistance index (PVRI) was above 5 Wood units m^2^. In Fontan patients with ventricular dysfunction or atrioventricular valve regurgitation, systemic VAD efficiently improves the hemodynamics. In Fontan patients with PVRI of > 5 Wood units m^2^, systemic VAD seems ineffective.

**Supplementary Information:**

The online version contains supplementary material available at 10.1186/s12576-024-00946-z.

## Introduction

Since the Fontan palliation was introduced in 1968 [1], the Fontan procedure has been the goal of surgical palliation in patients with a functional single ventricle. Despite remarkable improvements in early survival [2], their quality of life and life expectancy after Fontan procedure are unsatisfactory because of the multiple organ system dysfunction that often occurs in the long term [3]. Fontan circulation may collapse due to various reasons, such as systolic and/or diastolic ventricular dysfunction, atrioventricular value regurgitation and the elevation of pulmonary vascular resistance [4, 5]. In the acutely failing Fontan circulation, mechanical circulatory support is required, but it has still been a challenging issue in Fontan patients. Although several case reports demonstrated success with a right ventricular assist device (VAD) [6], the right ventricular support can be anatomically difficult because the need to disconnect the cavopulmonary connection to insert a VAD [7]. On the other hand, the introduction of systemic (left-sided) ventricular assist is technically easier even in the Fontan patients. However, the appropriate situation for the systemic ventricular assist in the Fontan circulation has remains controversial.

There have been several simulation studies focusing on the Fontan circulation. However, previous computational simulation studies of VAD in the Fontan circulation have mainly focused on the modes and patterns of VAD. Pekkan et al. tested four different pumps and three anatomical pathologies of the total cavopulmonary connection [8]. De Molfetta analyzed the effects of continuous and pulsatile flow VAD on Fontan patients [9]. They also tested the effects of the left and right ventricular and bi-ventricular assist devices in the Fontan circulation [10].

On the other hand, there are little studies focusing on patients’ pathophysiology. Therefore, in this study, we perform a computational simulation using a lumped parameter model to clarify which patients are acceptable for systemic ventricular assist therapy alone.

## Materials and methods

**Fig. 1:**
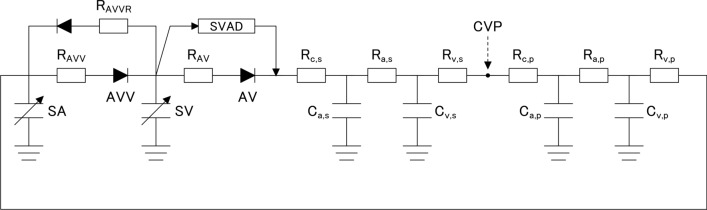
Lumped parameter model of the Fontan circulation with systemic ventricular assist device. *SA* single atrium, *SV* single ventricle, *AVV* atrioventricular valve, *AV* aortic valve, *SVAD* systemic ventricular assist device, *CVP* central venous pressure. *R*_AVV_, *R*_AVVR_ and *R*_AV_ donate resistances of atrioventricular valve, atrioventricular valve regurgitation and aortic valve, respectively. *R*_c,s_ and *R*_c,p_ donate characteristic impedances of systemic and pulmonary circulation, respectively. *R*_a,s_ and *R*_a,p_ donate systemic and pulmonary arterial resistance, respectively. *R*_v,s_ and *R*_v,p_ donate systemic and pulmonary venous resistances, respectively. *C*_a,s_ and *C*_p,s_ donate systemic and pulmonary arterial capacitances, respectively. *C*_v,s_ and *C*_v,p_ donate systemic and pulmonary venous capacitances, respectively

The electrical analog used to simulate the cardiovascular system of the Fontan circulation with a systemic VAD is shown in Fig. 1. Details of the simulation model have been described previously [11–14]. In this study, an adult man with a body surface area of 1.9 m^2^ was simulated.

### Heart

The ventricular and atrial chambers are represented by a time-varying elastance model [11–14]. The pressure and volume of each chamber are related by

1 $${P}_{\text{cc}}\left(t\right)=\left[{P}_{\text{es},\text{cc}}\left({V}_{\text{cc}}\right)-{P}_{\text{ed},\text{cc}}\left({V}_{\text{cc}}\right)\right]{e}_{\text{cc}}\left(t\right)+{P}_{\text{ed},\text{cc}}\left({V}_{\text{cc}}\right),$$

2 $${P}_{\text{ed},\text{cc}}={A}_{\text{cc}}\left[{e}^{{B}_{\text{cc}}\left({V}_{\text{ed},\text{cc}}-{V}_{0,\text{cc}}\right)}-1\right],$$

3 $${P}_{\text{es},\text{cc}}={E}_{\text{es},\text{cc}}\left[{V}_{\text{es},\text{cc}}-{V}_{0,\text{cc}}\right],$$

4 $${e}_{\text{cc}}\left(t\right)=\left\{\begin{array}{cc}0.5\left[1-\text{cos}\left(\pi t/{T}_{\text{es},\text{cc}}\right)\right]& 0\le t<{2T}_{\text{es},\text{cc}}\\ 0& {2T}_{\text{es},\text{cc}}\le t<{T}_{\text{c}}\end{array},\right.$$

where *P*_cc_ and *V*_cc_ are the chamber pressure and volume, respectively [cc denotes single atrial (SA) or single ventricular (SV) chamber], and t is the time from the start of systole. The chamber pressure is modeled as the sum of the end-diastolic pressure (*P*_ed,cc_ Eq. 2) and developed pressure [difference between end-systolic pressure (*P*_es,cc_, Eq. 3) and *P*_ed,cc_] scaled by the normalized elastance [*e*_cc_(*t*), Eq. 4]. The baseline parameters used in this model are listed in Table 1. Each valve was represented as an ideal diode connected serially to a small resistor (*R*_AV_, aortic: *R*_AVV_, atrioventricular). For patients with atrioventricular valve regurgitation, regurgitation is modeled as a reverse-connected resistor (*R*_AVVR_) and diode (see Fig. 1).

**Table 1:** Parameter used in the Fontan circulation and ventricular assist device models

Heart rate, beats/min	75	
Duration of cardiac cycle (T_c_), ms	800	
Time advance of atrial systole, ms	16	
	SV	SA
Time to end systole (T_es,cc_), ms	200	120
End-systolic elastance (E_es,cc_), mmHg/mL	3	0.5
Scaling factor of EDPVR (A_cc_), mmHg	0.35	0.06
Exponent for EDPVR (B_cc_), mL^-1^	0.033	0.264
Unstressed volume (V_0,cc_), mL	0	5
Aortic valvular resistance (forward) (R_AV_), mmHg s mL^-1^	0.001	
Atrioventricular valvular resistance (forward) (R_AVV_), mmHg s mL^-1^	0.001	
	Systemic (_s_)^a^	Pulmonary (_p_)^b^
Arterial resistance (R_a_), mmHg s mL^-1^	0.7	0.03
Characteristic impedance (R_c_), mmHg s mL^-1^	0.03	0.02
Venous resistance (R_v_), mmHg s mL^-1^	0.015	0.015
Arterial capacitance (C_a_), mL/mmHg	1.32	13
Venous capacitance (C_v_), mL/mmHg	70	8
K_A_, mmHg/rpm^2^	3.45×10^-6^	
K_B_, mmHg L/min/rpm	-5.9×10^-5^	
K_C_, mmHg L^2^/rpm^2^	-1.45	

cc denotes single ventricular (SV) or single atrial (SA) chamber. EDPVR, endo-diastolic pressure–volume relation

^a^For each variable, the systemic circulation is denoted by adding the subscript (_s_, such as *R*_a,s_)

^b^For each variable, the pulmonary circulation is denoted by adding the subscript (_p_, such as *R*_a,p_)

### Vascular system

Pulmonary and systemic vascular systems were modeled as modified three-element Windkessel models (Fig. 1). Each vascular system is modeled by lumped venous (*C*_v_) and arterial (*C*_a_) capacitances, characteristic impedance (*R*_c_), arterial resistance (*R*_a_), and resistance proximal to *C*_v_ (*R*_v_). For each variable, the pulmonary circulation is denoted by adding the subscript (_p_, such as *R*_a,p_), and the systemic circulation is denoted by adding the subscript (_s_, such as *R*_a,s_). The linear relationship between the pressure drops (Δ*P*) and flows (*Q*) in each resistance (*R*) (Eq. 5), the relationship between the pressure (*P*_c_) and volume (*V*_c_) in each capacitance *C* (Eq. 6), and the change in volume in each capacitance [d*V*(*t*)/d*t*] calculated by the difference between the inflow (*Q*_inflow_) and outflow (*Q*_ouflow_) (Eq. 7) was used to describe each vascular system:

5 $$\Delta P=QR,$$

6 $${P}_{\text{c}}=\frac{{V}_{\text{c}}}{C},$$

7 $$\frac{\text{d}V(t)}{\text{d}t}=\sum {Q}_{\text{inflow}}(t)-\sum {Q}_{\text{outflow}}\left(t\right),$$

where *Q*_inflow_(*t*) and *Q*_outflow_(*t*) are volumetric inflow and outflow, respectively.

### Total stressed blood volume

The total stressed blood volume (*V*_s_) is defined as the sum of the stressed volumes in all capacitances and chambers:

8 $${V}_{\text{s}}={V}_{\text{SA}}+{V}_{\text{SV}}+{V}_{{C}_{\text{a},\text{s}}}+{V}_{{C}_{\text{v},\text{s}}}+{V}_{{C}_{\text{a},\text{p}}}+{V}_{{C}_{\text{v},\text{p}}}.$$

### Rotational pump model

The flow of a rotational pump was described as a function of the pressure head (Δ*P*) and rotational frequency (*r*) in a previous study [15]. Therefore, in this study, a nonlinear function was used to simulate the flow of the rotational pump (*Q*_pump_):

9 $$\Delta P={K}_{\text{A}}\cdot {r}^{2}+{K}_{\text{B}}\cdot r{\cdot Q}_{\text{pump}}+{K}_{\text{C}}\cdot {\left({Q}_{\text{pump}}\right)}^{2}.$$

**Fig. 2:**
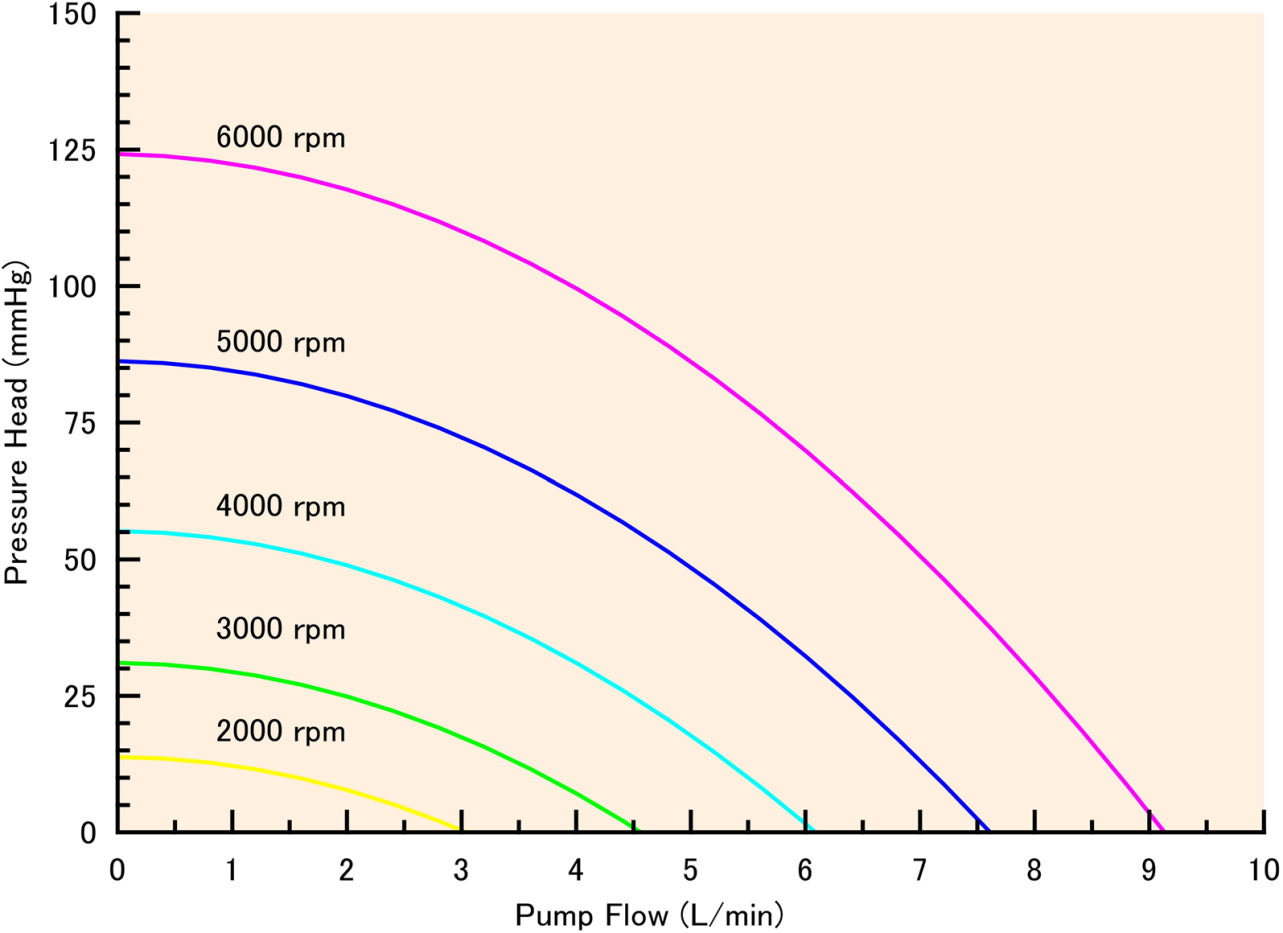
Non-liner relation between pump flow and pressure head at 2000, 3000, 4000, 5000 and 6000 rpm. This flow-pressure relation is almost similar to that of HeartMate III (Abbott)

This flow characteristics is similar to that of HeartMate III (Abbott, Abbott Park, IL, USA) (Fig. 2) [16].

Simultaneous differential equations (Eqs. 1–9) were solved using MATLAB/Simulink R2022a (MathWorks, Natick, MA, USA).

### Protocols

#### The effect of systemic VAD in a variety of pathophysiology

First, four types of failing Fontan circulation models—systolic ventricular dysfunction, diastolic ventricular dysfunction, atrioventricular valve regurgitation, and high pulmonary vascular resistance model—were simulated. Because mild elevations in pulmonary vascular resistance index (PVRI) coupled with low cardiac index identify patients at high risk of Fontan failure [17], the PVRI was set at 3 Wood units· m^2^ [*R*_a,p_ = 0.06; *R*_v,p_ = 0.015; *R*_c,p_ = 0.02 (mmHg s mL^−1^)] except for the high pulmonary vascular resistance model. Because Fahnhorst et al. reported that median central venous pressure before VAD placement was 20 mmHg (IQR, 17.75–22.25 mmHg) [18], the parameters of each model were adjusted to simulate failing Fontan circulation with a cardiac index of 1.9 L/min^/^m^2^ and a central venous pressure of 20 mmHg. Central venous pressure (CVP) was defined as a pressure distal to *R*_v,s_ (Fig. 1) and calculated by the following equation:

$$\text{CVP}=\frac{{P}_{{C}_{\text{a},\text{p}}}\cdot {R}_{\text{v},\text{s}}+{P}_{{C}_{\text{v},\text{s}}}\cdot {R}_{\text{c},\text{p}}}{{R}_{\text{v},\text{s}}+{R}_{\text{c},\text{p}}},$$

where *P*_Cv,s_ and *P*_Ca,p_ were the pressures in capacitances *C*_v,s_ and *C*_a,p,_ respectively.

1. In the systolic ventricular dysfunction model (Table S1), *E*_es,sv_ and stressed blood volume were adjusted to 1.215 mmHg/mL and 2036 mL, respectively.
2. In the diastolic ventricular dysfunction model (Table S2), *B*_sv_　and stressed blood volume were adjusted to 0.0511 mL^−1^ and 1999 mL, respectively.
3. In the atrioventricular valve regurgitation model (Table S3), *R*_AVVR_ and stressed blood volume were adjusted to 0.225 mmHg s mL^−1^ and 2015 mL, respectively.
4. In the high pulmonary vascular resistance model (Table S4), *R*_a,p_ and stressed blood volume were adjusted to be 0.23 mmHg s mL^−1^ and 1903 mL, respectively.

Second, in each failing Fontan circulation model, systemic VAD was instituted from the single ventricle to the aorta, and the rotational frequency was increased from 3000 to 4000 rpm. At a rotational frequency of 4000 rpm, the stressed blood volume was drawn stepwise in decrements of 100 mL until the mean pressure or volume of each chamber reached approximately zero, but not the minimum pressure or volume below zero. If the pressure or volume of each chamber reached below zero after VAD initiation, the stressed blood volume was increased stepwise in increments of 100 mL until the pressure or volume reached above zero. When the stressed blood volume was varied, the initial volume for systemic venous capacitance was decreased or increased and the simulation was started. After several dozen beats of simulation, the steady-state hemodynamic values were collected.

#### Effect of pulmonary vascular resistance on systemic VAD

*R*_a,p_ was increased stepwise from 0.06 to 0.25 mmHg s mL^−1^ (PVRI:3 to 9 Wood units· m^2^) in a systolic and diastolic ventricular dysfunction model (*E*_es,sv_ = 1.215 mmHg/mL, *B*_sv_ = 0.0511 mL^−1^) under systemic ventricular assist at a rotational frequency of 3500 rpm to evaluate the effect of elevated pulmonary vascular resistance. The stressed blood volume was controlled to achieve a mean pressure of approximately 0 mm Hg for a single atrium. Cardiac index, mean single atrial pressure, mean central venous pressure, and mean blood pressure were calculated for each PVRI.

## Results

**Fig. 3:**
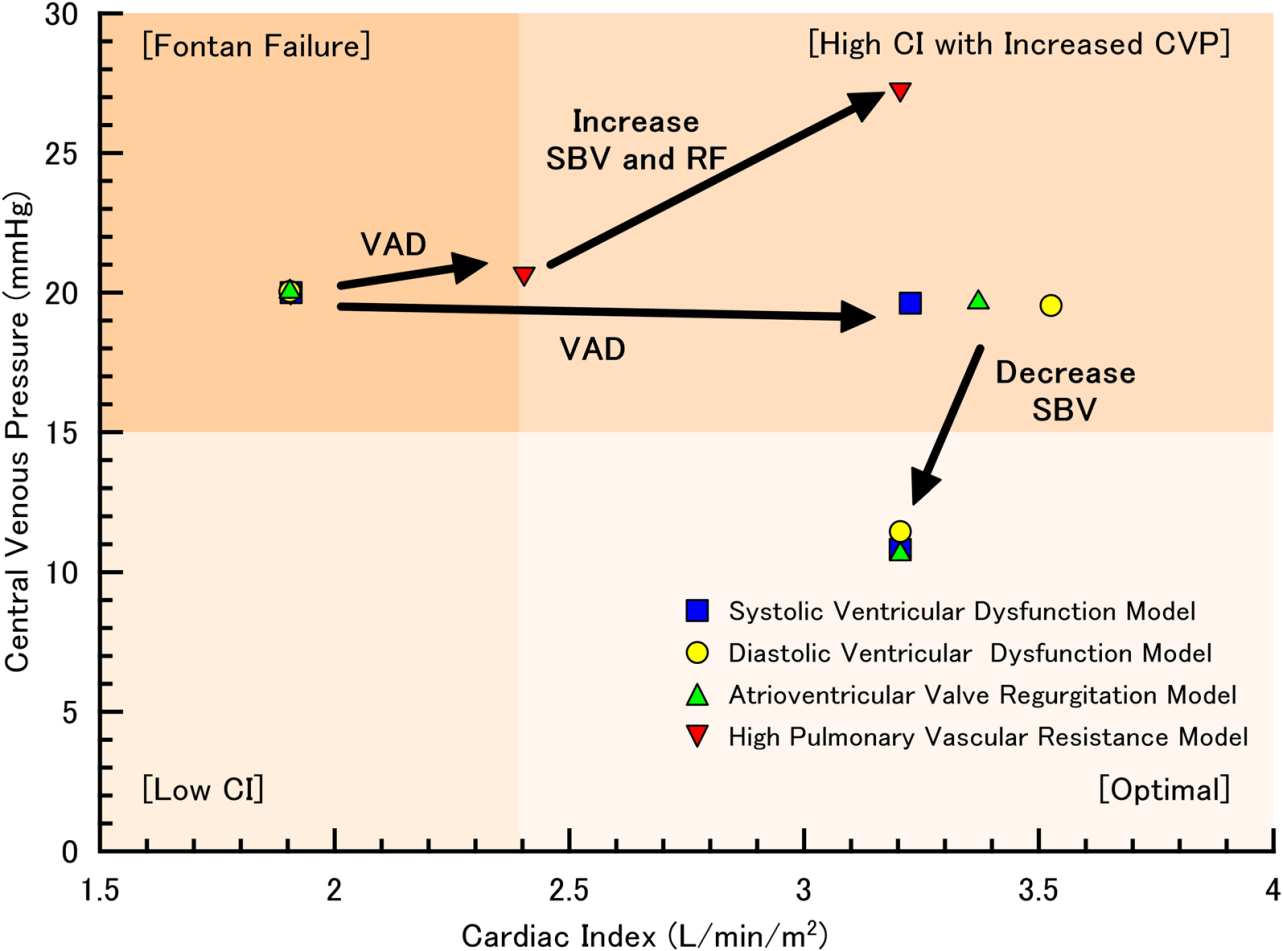
Ration between cardiac index and central venous pressure before and after ventricular assist. First, ventricular assist device (VAD) was introduced without altering stressed blood volume (SBV). The rotational frequency (RF) of VAD was set at 4000 rpm in systolic and diastolic dysfunction models and atrioventricular valve regurgitation model and at 3000 rpm in high pulmonary vascular resistance model. Next, The RF of VAD was set at 4000 rpm and then the SBV was adjusted in increments or decrements of 100 mL to become the mean single atrial pressure around zero, but not the minimum pressure below zero. Blue squares and yellow circles represent the simulation results in systolic and diastolic dysfunction models, respectively. Light-green triangles represent those in atrioventricular valve regurgitation model. Red inverted triangles represent those in high pulmonary vascular resistance model

When a systolic ventricular dysfunction model with *E*_es,sv_ of 1.215 mmHg/mL and stressed blood volume of 2036 mL was simulated, a cardiac index of 1.91 L/min/m^2^ and a systemic central venous pressure of 20.0 mmHg were achieved (Table 2.). Once the systemic VAD was initiated at a rotational frequency of 3000 rpm, the cardiac index increased to 2.89 L/min/m^2^. Central venous pressure was maintained at 19.7 mmHg. After increasing the rotational frequency to 4000 rpm, the cardiac index was 3.23 L/min/m^2^, and central venous pressure was maintained at 19.6 mmHg with single atrial and mean blood pressures of 9.92 mmHg and 95.7 mmHg, respectively (Fig. 3). When the stressed blood volume was reduced to 1136 mL (minus 900 mL), the single atrial pressure reached 1.18 mmHg, cardiac index was 3.20 L/min/m^2^, mean blood pressure was 86.4 mmHg, and central venous pressure was 10.8 mmHg (Fig. 3).

**Table 2.** Effect of systemic ventricular assist device in a variety of pathophysiology

*Systolic ventricular dysfunction*					
End-systolic elastance (E_es,sv_), mmHg/mL	1.215				
Stressed blood volume, mL	2036	2036	2036	2036	1136
Cardiac index, L/min/m^2^	1.91	2.89	3.05	3.23	3.20
Mean blood pressure, mmHg	65.0	87.8	91.7	95.7	86.4
Central venous pressure, mmHg	20.0	19.7	19.7	19.6	10.8
Single atrial pressure, mmHg	14.3	11.0	10.5	9.92	1.18
Rotational frequency, rpm		3000	3500	4000	4000
*Diastolic ventricular dysfunction*					
Exponent for EDPVR (B_sv_), mL^-1^	0.0511				
Stressed blood volume, mL	1999	1999	1999	1999	1199
Cardiac index, L/min/m^2^	1.90	3.13	3.33	3.53	3.20
Mean arterial blood pressure, mmHg	65.0	93.5	98.1	102.7	87.1
Central venous pressure, mmHg	20.0	19.7	19.6	19.5	11.5
Single atrial pressure, mmHg	14.3	10.2	9.58	8.93	1.82
Rotational frequency, rpm		3000	3500	4000	4000
*Atrioventricular valve regurgitation*					
Regurgitation resistance (R_AVVR_), mmHg s mL^-1^	0.225				
Stressed blood volume, mL	2015	2015	2015	2015	1115
Cardiac index, L/min/m^2^	1.90	3.00	3.18	3.37	3.20
Mean arterial blood pressure, mmHg	65.0	90.4	94.8	99.2	86.3
Central venous pressure, mmHg	20.0	19.7	19.7	19.6	10.6
Single atrial pressure, mmHg	14.3	10.7	10.1	9.51	1.00
Regurgitation fraction, %	50.9	41.1	39.6	38.0	2.00
Rotational frequency, rpm		3000	3500	4000	4000
*High pulmonary vascular resistance*					
Pulmonary arterial resistance (R_a,p_), mmHg s mL^-1^	0.230				
Stressed blood volume, mL	1903	1903	2203	2503	
Cardiac index, L/min/m^2^	1.91	2.40	2.80	3.20	
Mean arterial blood pressure, mmHg	65.0	77.4	90.1	102.9	
Systemic central venous pressure, mmHg	20.0	20.7	24.0	27.3	
Single atrial pressure, mmHg	4.01	0.506	0.445	0.382	
Rotational frequency, rpm		3000	3500	4000

When a diastolic ventricular dysfunction model with *B*_sv_ of 0.0511 mL^−1^ and stressed volume of 1999 mL was simulated, cardiac index was 1.90 L/min/m^2^ and systemic central venous pressure was 20.0 mmHg (Table 2.). Once the systemic VAD was initiated at a rotational frequency of 3000 rpm, the cardiac index increased to 3.13 L/min/m^2^ and central venous pressure was maintained at 19.7 mmHg. After increasing the rotational frequency to 4000 rpm, the cardiac index was 3.53 L/min/m^2^ and central venous pressure remained at 19.5 mmHg (Fig. 3). The single atrial and mean blood pressures were 8.93 mmHg and 102.7 mmHg, respectively. When the stressed blood volume was reduced to 1199 mL (minus 900 mL) in this model, the single atrial pressure reached 1.82 mmHg. The cardiac index was 3.20 L/min/m^2^, mean blood pressure was 87.1 mmHg, and central venous pressure decreased to 11.5 mmHg (Fig. 3).

When an atrioventricular valve regurgitation model with R_AVVR_ of 0.225 mmHg s mL^−1^ and stressed blood volume of 2015 mL was simulated, the cardiac index was 1.90 L/min/m^2^, the central venous pressure was 20.0 mmHg, and the regurgitation fraction was 50.9% (Table 3). Once the systemic VAD was initiated at a rotational frequency of 3000 rpm, the cardiac index increased to 3.00 L/min/m^2^, and central venous pressure was maintained at 19.7 mmHg. After increasing the rotational frequency to 4000 rpm, the cardiac index was 3.37 L/min/m^2^, and central venous pressure remained at 19.6 mmHg with single atrial and mean blood pressures of 9.51 mmHg and 99.2 mmHg, respectively (Fig. 3). When the stressed blood volume was reduced to 1115 mL (minus 900 mL), the single atrial pressure was 1.00 mmHg, cardiac index was 3.20 L/min/m^2^, mean blood pressure was 86.3 mmHg, and central venous pressure was 10.6 mmHg (Fig. 3).

**Table 3:** Effect of pulmonary vascular resistance in patients with systemic ventricular assist device

Pulmonary vascular resistance index, Woods units m^2^	3.01	3.96	5.07	6.02	6.97	8.08	9.03
Pulmonary arterial resistance (*R*_a,p_), mmHg s mL^−1^	0.06	0.09	0.125	0.155	0.185	0.22	0.25
End-systolic elastance (*E*_es,sv_), mmHg/mL	1.215						
Exponent for EDPVR (*B*_sv_), mL^−1^	0.0511						
Rotational frequency, rpm	3500						
Stressed blood volume, mL	910	1135	1397	1621	1846	2108	2332
Cardiac index, L/min/m^2^	2.80	2.80	2.80	2.80	2.80	2.80	2.80
Mean arterial blood pressure, mmHg	74.9	77.5	80.6	83.3	86.0	89.1	91.7
Central venous pressure, mmHg	8.70	11.4	14.5	17.1	19.8	22.9	25.6
Single atrial pressure, mmHg	0.260	0.263	0.262	0.256	0.260	0.259	0.253

In a high pulmonary vascular resistance model with *R*_a,p_ of 0.230 mmHg s mL^−1^ (PVRI = 8.39 Wood units m^2^) and stressed blood volume of 1903 mL, cardiac index was 1.91 L/min/m^2^ and central venous pressure was 20.0 mmHg (Table 2.). Once systemic VAD was initiated at a rotational frequency of 3000 rpm, the cardiac index and central venous pressure increased to 2.40 L/min/m^2^ and 20.7 mmHg, respectively. To increase the rotational frequency above 3000 rpm, an additional stressed blood volume was required in this model. At a rotational frequency of 4000 rpm, an additional stressed blood volume of 600 mL was required to maintain the pressure or volume of each chamber above zero. Then, cardiac index became 3.20 L/min/m^2^, the mean blood pressure was 102.9 mmHg, and the central venous pressure was increased to 27.3 mmHg (Fig. 3).

**Fig. 4:**
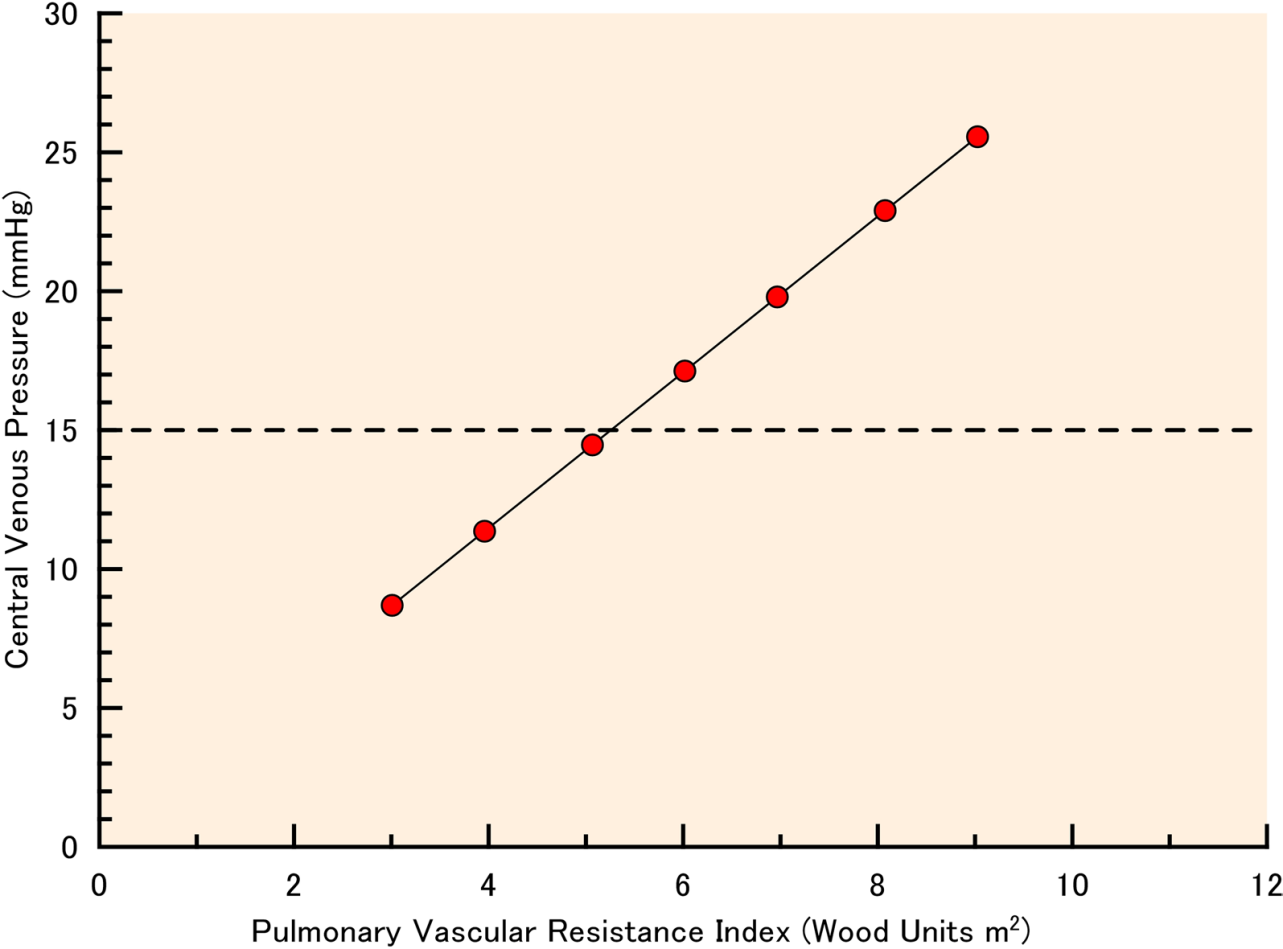
Relation between pulmonary vascular resistance index and central venous pressure. When pulmonary vascular resistance index (PVRI) was varied from 3 to 9 Wood units m^2^, the rotational frequency of ventricular assist device was set at 3500 rpm and the stressed blood volume was adjusted in increments of 1 mL to become the mean single atrial pressure around zero, but not the minimum pressure below zero. With an increase in PVRI, the central venous pressure rapidly increased. If we define acceptable central venous pressure as less than 15 mmHg, systemic ventricular assist tolerates PVRI less than 5 Wood units m^2^

Finally, we simulated the model in which pulmonary vascular resistance was varied. With the stepwise increase in *R*_a,p_, the PVRI increased from 3 to 9 wood units m^2^. Stressed blood volume was controlled to maintain a mean single atrial pressure of approximately 0 mmHg, but not the minimum pressure below 0 mmHg. When the PVRI was 3 Wood units m^2^, the stressed blood volume was 910 mL. Higher PVRI required larger stressed blood volume to maintain the single atrial pressure above 0 mmHg. An additional stressed blood volume of 1422 mL was required to maintain the single atrial pressure above 0 mmHg when the PVRI was 9 Wood units m^2^ compared with that at a PVRI of 3 Wood units m^2^. A higher PVRI required more stressed blood volume, resulting in a remarkable increase in central venous pressure (Table 3; Fig. 4).

## Discussion

In this study, four types of failure pathophysiology, systolic and diastolic ventricular dysfunction, atrioventricular valve regurgitation and elevated pulmonary vascular resistance, were simulated to evaluate the efficacy of systemic VAD on the Fontan circulation. This study demonstrated that systemic VADs are effective in patients with systolic and/or diastolic ventricular dysfunction, atrioventricular valve dysfunction, or even with elevated pulmonary vascular resistance when PVRI is less than 5 Wood units·m^2^.

### Systolic ventricular dysfunction

In the first few decades after the Fontan procedure, systolic ventricular function is usually preserved but declines over time [19, 20]. Systolic ventricular dysfunction after the Fontan procedure has been reported to be associated with high mortality [21]. Fahnhorst et al. demonstrates the effectiveness of the single VAD in the Fontan patients with systolic ventricular dysfunction [18]. In this study, the initiation of a systemic VAD remarkably increased the cardiac index and blood pressure. At the time of initiating systemic VAD, the decrease in central venous pressure was less than 0.5 mmHg. After reducing stressed blood volume, central venous pressure achieved an acceptable value of 10.8 mmHg in this model. Then, cardiac index and mean blood pressure were maintained at 3.20 L/min/m^2^ and 86.4 mmHg, respectively, and this demonstrates that the cardiac index and blood pressure increased without a decrease in central venous pressure once the systemic VAD was initiated. Stressed blood volume reduction is necessary to decrease central venous pressure while maintaining an adequate cardiac index and mean blood pressure. Therefore, in clinical settings, systemic VAD implantation enables a decrease in central venous pressure by dehydrating patients with the use of diuretics or hemodialysis, as the pathophysiology of this condition is characterized by maintaining cardiac output by increasing the stressed blood volume.

### Diastolic ventricular dysfunction

A substantial number of patients with Fontan circulation have diastolic ventricular dysfunction [22, 23]. The use of systemic VADs for diastolic ventricular dysfunction in absence of systolic failure remains controversial. In this study, diastolic ventricular dysfunction model showed a similar trend to the systolic ventricular dysfunction model. The initiation of systemic VAD remarkably increased the cardiac index and blood pressure without decreasing the central venous pressure. After stressed blood volume reduction, central venous pressure dropped with adequate cardiac index (3.20 L/min/m^2^) and mean blood pressure (87.1 mmHg). Therefore, reduction of stressed blood volume is possible in Fontan patients with diastolic ventricular dysfunction who have systemic VAD support to achieve a lower central venous pressure.

### Atrioventricular valve regurgitation

More than moderate atrioventricular valve regurgitation is a significant risk factor for long-term mortality in Fontan patients [24]. Most patients experience valve failure during the first 30 years of life [25]. Significant atrioventricular regurgitation compromises Fontan circulation due to volume overload, ventricular dilatation, reduced ventricular function, and increased postcapillary and central venous pressures. Although a variety of techniques have been described for valve repair, attaining a competent valve is often challenging, and valve replacement is inevitable in some cases [26, 27]. Cedars et al. reported that 65% of Fontan patients implanted with a VAD had moderate or severe atrioventricular regurgitation [28]. In the atrioventricular valve regurgitation model, systemic VAD is also effective, but a reduction in the stressed blood volume is necessary to achieve an acceptable low central venous pressure. A question may arise whether valve repair is necessary when systemic VAD is established. In fact, Nandi et al. reported a technique for atrioventricular valve excision in continuous-flow ventricular assist device implantation [29]. However, findings of this study suggest that valve repair is unnecessary with the support of a systemic VAD. Therefore, atrioventricular valve regurgitation itself may not impede systemic VAD implantation.

### Elevated pulmonary vascular resistance

In the absence of a subpulmonary pump, increased pulmonary vascular resistance is critical, as it causes upstream congestion and decreased downstream flow. In pump failure and valve failure models (systolic ventricular dysfunction, diastolic ventricular dysfunction, and atrioventricular valve regurgitation), this study demonstrated significant hemodynamic improvement under moderately high pulmonary vascular resistance (PVRI = 3.01 Wood units· m^2^). However, systemic VAD did not work well in a remarkably high pulmonary vascular resistance model with an *R*_a,p_ of 0.230 mmHg s mL^−1^ (PVRI = 8.39 Wood units·m^2^), in which a large additional stressed blood volume was required to increase the rotational frequency. One of the most important clinical questions is how systemic VAD augments cardiac output in high pulmonary vascular resistance. In our model, with a stepwise increase in PVRI, the cardiac index was maintained at more than 2.2 L/min/m^2^, meaning no hypoperfusion in the Forrester’s subset. On the other hand, the central venous pressure remarkably increased with an increase in PVRI. If we define acceptable central venous pressure as less than 15 mmHg, systemic VAD tolerates PVRI less than 5 Wood units m^2^ (Fig. 4). When the patients’ PVRI is more than 5 Wood units m^2^, the use of sub-pulmonary VAD may be required.

### Limitations

This study had several methodological limitations. First, the parameters used in the present model were fixed, except for a few parameters. A change in *R*_a,p_ may affect other parameters, such as *C*_a,p_ in the clinical setting. Further experimental and clinical implications are necessary to include interactions among the parameters in our simulation. Second, most patients with failing Fontan circulation have fenestrations or shunts, which were excluded in this model. These factors should affect the simulation results. In this study, we simplified the model to examine the effects of systemic VAD in patients with Fontan circulation. Because of the complexity of this circulation, it may be essential to simulate each patient using this method to understand the possible effects of systemic VAD. Third, venous systems have non-linearities, but non-linearities are omitted in the modified three-element Windkessel vasculature model. Because Mehlsen has reported that non-linearities in pressure/flow relationships need to be considered in low-flow conditions [30], the non-linearities of the venous systems may affect the simulation results, especially in the low-flow conditions of Fontan failure.

## Conclusion

In the three models of Fontan failure (systolic ventricular, diastolic ventricular, or atrioventricular valve dysfunction), systemic VAD effectively increased the cardiac index and blood pressure. With an adequate reduction in the stressed blood volume, systemic VAD can decrease central venous pressure. In contrast, in Fontan failure with increased PVRI, central venous pressure increased even under systemic VAD support. Although further clinical investigations are needed, in patients with extremely high pulmonary vascular resistance of over 5 Wood units·m^2^, systemic VAD may be less effective, and sub-pulmonary VAD or biventricular VAD should be discussed.

## Supplementary Information

Supplementary Material 1.

## Data Availability

The data sets used and analyzed during the current study and source codes are available from the corresponding author on reasonable request.

## Abbreviations

PVRI: Pulmonary vascular resistance index
SA: Single atrium
SV: Single ventricle
VAD: Ventricular assist device
