# Supplementary material for: Effects of systemic ventricular assist in failing Fontan patients: a theoretical analysis using a computational model
Source: J Physiol Sci. 2024 Nov 2;74:53. doi: 10.1186/s12576-024-00946-z (PMC11531161; doi:10.1186/s12576-024-00946-z)
Supplement: Supplementary file 1 — Supplementary Material 1. [file 12576_2024_946_MOESM1_ESM.docx]

**Supplementary Tables**

**Table S1 Parameters used in the systolic ventricular dysfunction model.**

| Heart rate, beats/min | 75 |  |
| --- | --- | --- |
| Duration of cardiac cycle (T_c_), ms | 800 |  |
| Time advance of atrial systole, ms | 16 |  |
|  | SV | SA |
| Time to end systole (T_es,cc_), ms | 200 | 120 |
| End-systolic elastance (E_es,cc_), mmHg/mL | 1.215 | 0.5 |
| Scaling factor of EDPVR (A_cc_), mmHg | 0.35 | 0.06 |
| Exponent for EDPVR (B_cc_), mL^-1^ | 0.033 | 0.264 |
| Unstressed volume (V_0,cc_), mL | 0 | 5 |
| Aortic valvular resistance (forward) (R_AV_), mmHg s mL^-1^ | 0.001 |  |
| Atrioventricular valvular resistance (forward) (R_AVV_), mmHg s mL^-1^ | 0.001 |  |
|  | Systemic (_s_)^a^ | Pulmonary (_p_)^b^ |
| Arterial resistance (R_a_), mmHg s mL^-1^ | 0.7 | 0.06 |
| Characteristic impedance (R_c_), mmHg s mL^-1^ | 0.03 | 0.02 |
| Venous resistance (R_v_), mmHg s mL^-1^ | 0.015 | 0.015 |
| Arterial capacitance (C_a_), mL/mmHg | 1.32 | 13 |
| Venous capacitance (C_v_), mL/mmHg | 70 | 8 |
| K_A_, mmHg/rpm^2^ | 3.45×10^-6^ |  |
| K_B_, mmHg L/min/rpm | -5.9×10^-5^ |  |
| K_C_, mmHg L^2^/rpm^2^ | -1.45 |  |
| cc denotes single ventricular (SV) or single atrial (SA) chamber. EDPVR, endo-diastolic pressure-volume relation | | |
| ^a^For each variable, the systemic circulation is denoted by adding the subscript (_s_, such as R_a,s_) | | |
| ^b^For each variable, the pulmonary circulation is denoted by adding the subscript (_p_, such as R_a,p_) | | |

**Table S2 Parameters used in the diastolic ventricular dysfunction model.**

| Heart rate, beats/min | 75 |  |
| --- | --- | --- |
| Duration of cardiac cycle (T_c_), ms | 800 |  |
| Time advance of atrial systole, ms | 16 |  |
|  | SV | SA |
| Time to end systole (T_es,cc_), ms | 200 | 120 |
| End-systolic elastance (E_es,cc_), mmHg/mL | 3 | 0.5 |
| Scaling factor of EDPVR (A_cc_), mmHg | 0.35 | 0.06 |
| Exponent for EDPVR (B_cc_), mL^-1^ | 0.0511 | 0.264 |
| Unstressed volume (V_0,cc_), mL | 0 | 5 |
| Aortic valvular resistance (forward) (R_AV_), mmHg s mL^-1^ | 0.001 |  |
| Atrioventricular valvular resistance (forward) (R_AVV_), mmHg s mL^-1^ | 0.001 |  |
|  | Systemic (_s_)^a^ | Pulmonary (_p_)^b^ |
| Arterial resistance (R_a_), mmHg s mL^-1^ | 0.7 | 0.06 |
| Characteristic impedance (R_c_), mmHg s mL^-1^ | 0.03 | 0.02 |
| Venous resistance (R_v_), mmHg s mL^-1^ | 0.015 | 0.015 |
| Arterial capacitance (C_a_), mL/mmHg | 1.32 | 13 |
| Venous capacitance (C_v_), mL/mmHg | 70 | 8 |
| K_A_, mmHg/rpm^2^ | 3.45×10^-6^ |  |
| K_B_, mmHg L/min/rpm | -5.9×10^-5^ |  |
| K_C_, mmHg L^2^/rpm^2^ | -1.45 |  |
| cc denotes single ventricular (SV) or single atrial (SA) chamber. EDPVR, endo-diastolic pressure-volume relation | | |
| ^a^For each variable, the systemic circulation is denoted by adding the subscript (_s_, such as R_a,s_) | | |
| ^b^For each variable, the pulmonary circulation is denoted by adding the subscript (_p_, such as R_a,p_) | | |

**Table S3 Parameters used in the atrioventricular valve regurgitation model.**

| Heart rate, beats/min | 75 |  |
| --- | --- | --- |
| Duration of cardiac cycle (T_c_), ms | 800 |  |
| Time advance of atrial systole, ms | 16 |  |
|  | SV | SA |
| Time to end systole (T_es,cc_), ms | 200 | 120 |
| End-systolic elastance (E_es,cc_), mmHg/mL | 3 | 0.5 |
| Scaling factor of EDPVR (A_cc_), mmHg | 0.35 | 0.06 |
| Exponent for EDPVR (B_cc_), mL^-1^ | 0.033 | 0.264 |
| Unstressed volume (V_0,cc_), mL | 0 | 5 |
|  | | |
| Aortic valvular resistance (forward) (R_AV_), mmHg s mL^-1^ | 0.001 |  |
| Atrioventricular valvular resistance (forward) (R_AVV_), mmHg s mL^-1^ | 0.001 |  |
| (regurgitation) (R_AVVR_), mmHg s mL^-1^ | 0.225 |  |
|  | Systemic (_s_)^a^ | Pulmonary (_p_)^b^ |
| Arterial resistance (R_a_), mmHg s mL^-1^ | 0.7 | 0.06 |
| Characteristic impedance (R_c_), mmHg s mL^-1^ | 0.03 | 0.02 |
| Venous resistance (R_v_), mmHg s mL^-1^ | 0.015 | 0.015 |
| Arterial capacitance (C_a_), mL/mmHg | 1.32 | 13 |
| Venous capacitance (C_v_), mL/mmHg | 70 | 8 |
| K_A_, mmHg/rpm^2^ | 3.45×10^-6^ |  |
| K_B_, mmHg L/min/rpm | -5.9×10^-5^ |  |
| K_C_, mmHg L^2^/rpm^2^ | -1.45 |  |
| cc denotes single ventricular (SV) or single atrial (SA) chamber. EDPVR, endo-diastolic pressure-volume relation | | |
| ^a^For each variable, the systemic circulation is denoted by adding the subscript (_s_, such as R_a,s_) | | |
| ^b^For each variable, the pulmonary circulation is denoted by adding the subscript (_p_, such as R_a,p_) | | |

**Table S4 Parameters used in the high pulmonary vascular resistance model.**

| Heart rate, beats/min | 75 |  |
| --- | --- | --- |
| Duration of cardiac cycle (T_c_), ms | 800 |  |
| Time advance of atrial systole, ms | 16 |  |
|  | SV | SA |
| Time to end systole (T_es,cc_), ms | 200 | 120 |
| End-systolic elastance (E_es,cc_), mmHg/mL | 3 | 0.5 |
| Scaling factor of EDPVR (A_cc_), mmHg | 0.35 | 0.06 |
| Exponent for EDPVR (B_cc_), mL^-1^ | 0.033 | 0.264 |
| Unstressed volume (V_0,cc_), mL | 0 | 5 |
| Aortic valvular resistance (forward) (R_AV_), mmHg s mL^-1^ | 0.001 |  |
| Atrioventricular valvular resistance (forward) (R_AVV_), mmHg s mL^-1^ | 0.001 |  |
|  | Systemic (_s_)^a^ | Pulmonary (_p_)^b^ |
| Arterial resistance (R_a_), mmHg s mL^-1^ | 0.7 | 0.23 |
| Characteristic impedance (R_c_), mmHg s mL^-1^ | 0.03 | 0.02 |
| Venous resistance (R_v_), mmHg s mL^-1^ | 0.015 | 0.015 |
| Arterial capacitance (C_a_), mL/mmHg | 1.32 | 13 |
| Venous capacitance (C_v_), mL/mmHg | 70 | 8 |
| K_A_, mmHg/rpm^2^ | 3.45×10^-6^ |  |
| K_B_, mmHg L/min/rpm | -5.9×10^-5^ |  |
| K_C_, mmHg L^2^/rpm^2^ | -1.45 |  |
| cc denotes single ventricular (SV) or single atrial (SA) chamber. EDPVR, endo-diastolic pressure-volume relation | | |
| ^a^For each variable, the systemic circulation is denoted by adding the subscript (_s_, such as R_a,s_) | | |
| ^b^For each variable, the pulmonary circulation is denoted by adding the subscript (_p_, such as R_a,p_) | | |
